# Investigation of Radiation-induced Transcriptome Profile of Radioresistant Non-small Cell Lung Cancer A549 Cells Using RNA-seq

**DOI:** 10.1371/journal.pone.0059319

**Published:** 2013-03-22

**Authors:** Hee Jung Yang, Namshin Kim, Ki Moon Seong, HyeSook Youn, BuHyun Youn

**Affiliations:** 1 Department of Biological Sciences, Pusan National University, Busan, Republic of Korea; 2 Korean Bioinformation Center, Korea Research Institute of Bioscience and Biotechnology, Daejeon, Republic of Korea; 3 Division of Radiation Effect Research, Radiation Health Research Institute, Korea Hydro & Nuclear Power Co., Ltd., Seoul, Republic of Korea; 4 Department of Bioscience & Biotechnology, Institute of Bioscience, Sejong University, Seoul, Republic of Korea; National Taiwan University, Taiwan

## Abstract

Radioresistance is a main impediment to effective radiotherapy for non-small cell lung cancer (NSCLC). Despite several experimental and clinical studies of resistance to radiation, the precise mechanism of radioresistance in NSCLC cells and tissues still remains unclear. This result could be explained by limitation of previous researches such as a partial understanding of the cellular radioresistance mechanism at a single molecule level. In this study, we aimed to investigate extensive radiation responses in radioresistant NSCLC cells and to identify radioresistance-associating factors. For the first time, using RNA-seq, a massive sequencing-based approach, we examined whole-transcriptome alteration in radioresistant NSCLC A549 cells under irradiation, and verified significant radiation-altered genes and their chromosome distribution patterns. Also, bioinformatic approaches (GO analysis and IPA) were performed to characterize the radiation responses in radioresistant A549 cells. We found that epithelial–mesenchymal transition (EMT), migration and inflammatory processes could be meaningfully related to regulation of radiation responses in radioresistant A549 cells. Based on the results of bioinformatic analysis for the radiation-induced transcriptome alteration, we selected seven significant radiation-altered genes (*SESN2, FN1, TRAF4, CDKN1A, COX-2, DDB2* and *FDXR*) and then compared radiation effects in two types of NSCLC cells with different radiosensitivity (radioresistant A549 cells and radiosensitive NCI-H460 cells). Interestingly, under irradiation, *COX-2* showed the most significant difference in mRNA and protein expression between A549 and NCI-H460 cells. IR-induced increase of COX-2 expression was appeared only in radioresistant A549 cells. Collectively, we suggest that COX-2 (also known as prostaglandin-endoperoxide synthase 2 (PTGS2)) could have possibility as a putative biomarker for radioresistance in NSCLC cells.

## Introduction

Radiotherapy, alone or in combination with surgery or chemotherapy, plays a critical role in treatment of NSCLC. However, the therapeutic outcomes are not fully satisfactory in many cases. With unexpected radiation responses during radiotherapy, (intrinsic/acquired) radioresistance is considered as a main factor which restricts the efficacy of radiation treatment for NSCLC [1].

Radiosensitivity of cells and tissues has been linked directly to proliferation rates, and radiation-induced modifications of gene expression are mainly involved in cell cycle progression, DNA repair and apoptosis [2]. Radioresistance in NSCLC has been associated with loss of p53 function, altered expression of survival proteins such as X-linked inhibitor of apoptosis protein (XIAP) and survivin, activation of phosphoinositide 3-kinase (PI3K)/Akt signaling [3], or overexpression of Pim-1 kinase [4]. Also, accumulating evidence suggests that radioresistance is often correlated with epidermal growth factor receptor (EGFR) and overexpression of anti-oxidant enzymes such as Mn-superoxide dismutase (Mn-SOD) [5], [6]. Although these studies have contributed to understanding of the mechanisms for cellular radioresistance, they can explain only a partial aspect of radioresistant responses, and the comprehensive functional mechanisms remain largely elusive. This result is not surprising, considering the nature of radioresistance regulated by complex interactions between multiple genes and/or proteins. Moreover, there are several reports that radiosensitivity is not solely related to radiation-induced apoptosis, and may depend on additional molecules and processes that have not yet been identified [7]. Therefore, it has been difficult to elucidate the exact mechanism of radioresistance and to understand entire alteration of radiation responses in NSCLC.

Extensive gene expression profiling analysis can increase interpretation of the molecular mechanism for radioresistance modulated by complicated genetic and biochemical networks. Microarray is the most comprehensive approach to measure gene expression and has led to outstanding advances in knowledge of radioresistance [8], [9]. However, microarray has several limitations such as probe hybridization kinetics, probe selection (need to know genomic loci and features), background hybridization and cross-platform comparability [10]. Sequencing-based expression analysis has been developed to overcome these limitations in hybridization-based assay. Over the past 10 years, introduction of high-throughput next-generation sequencing (NGS) technologies have revolutionized transcriptomics by providing opportunities for multidimensional studies of cellular transcriptomes. It becomes possible because large-scale expression data are acquired at a single-base resolution. As a main quantitative transcriptome profiling platform, RNA-seq has been considered a new experimental method to replace microarray. In RNA-seq, (total or messenger) RNA population is converted to a library of cDNA fragments with adaptors attached to one or both ends. Each library, with or without amplification, is then sequenced to obtain short sequence reads from one end (single-end sequencing) or both ends (paired-end sequencing). The read sequences are typically 30–400 bp in length, depending on the sequencing platforms: Illumina, Roche 454 or SOLiD system. After sequencing, the resulting reads are either aligned to reference genome or transcripts, or assembled *de novo* without the genomic sequence [11]. Owing to high depth of read coverage, RNA-seq produces a more accurate measurement for levels of transcripts and their isoforms than other tools. Furthermore, it can be used to investigate transcript structures in context of transcription start sites, alternative splicing patterns and other post-transcriptional modifications. RNA-seq has been applied to identify long non-coding RNAs that play an important role in transcriptional and post-translational gene regulation [11].

Until now, there have been no studies to assess the global radiation responses at entire transcriptome level in NSCLC cells. We focused on RNA-seq to overcome the limitations of previous researches indicating somewhat fragmentary evidences of radioresponses, and then proposed that RNA-seq could be an ideal approach to gain insight into the complex radiation response. In this study, we aimed to characterize transcriptome of radioresistant NSCLC A549 cells and to investigate functional regulatory networks at the genetic level. To achieve these goals, we made full use of a strategic combination of the RNA-seq-derived gene expression data and the results of bioinformatic and biological assays. It is the first study to apply this emerging technology, RNA-seq to profile radiation-induced gene expression in radioresistant NSCLC cells. We suggest that our results could provide useful information for identification of potential biomarkers of radioresponses such as radioresistance, and ultimately help to understand the radiation effects in NSCLC cells.

## Materials and Methods

### Reagents

Cell culture media (RPMI 1640), FBS and antibiotics (penicillin and streptomycin) were acquired from Hyclone (Logan, UT). Antibodies against EGFR, p53, Sestrin2, COX-2, TRAF4 and β-actin were purchased from Santa Cruz Biotechnology (Santa Cruz, CA). Antibodies for phospho-EGFR (Tyr1068), phospho-EGFR (Tyr845), phospho-Akt (Ser473), phospho-Akt (Tyr308), Akt, phospho-p53 (Ser15), p21 and phosphatase and tensin homolog (PTEN) were acquired from Cell Signaling Technology (Beverly, MA). Antibody against γ-H2A.X (Ser139) was purchased from Millipore (Billerica, MA). Antibody for FDXR was purchased from Abcam (Austin, TX).

### Cell Culture and Irradiation

Human NSCLC cell lines (A549 and NCI-H460) were acquired from Korean Cell Line Bank (Seoul, Republic of Korea). Both cells were cultured in RPMI 1640 medium supplemented with penicillin (100 U/ml), streptomycin (100 U/ml) and 10% FBS. Cells were exposed to ionizing radiation (IR) using the ^137^Cs γ-irradiator (IBL 437C, CIS Bio International) at a dose rate of 0.8 Gy/min. Irradiated cells were incubated for an additional 4 hr.

### RNA-seq Library Preparation and Sequencing

Total RNA was isolated from irradiated and non-irradiated A549 cells using RNeasy Mini Kit (Qiagen, Valencia, CA). RNA quality was assessed by agarose gel electrophoresis (visual absence of significant 28S and 18S rRNA degradation) and by spectrophotometry. Next, total RNA integrity was checked using an Agilent Technologies 2100 Bioanalyzer with an RNA integrity number (RIN) value greater than 8. The mRNA was purified and fragmented from total RNA (2 µg) using poly-T oligo-attached magnetic beads with two rounds of purification. Cleaved RNA fragments primed with random hexamers were reverse transcribed into first-strand cDNA using reverse transcriptase and random primers. The RNA template was removed and synthesized a replacement strand to generate double-strand cDNA. End repair, A-tailing, adaptor ligation, cDNA template purification and enrichment of the purified cDNA templates using PCR were then performed. Constructed libraries were 100 bp paired-end sequenced by an Illumina GAIIx sequencer on two lanes of the flow cell, following manufacturer’s instructions.

### Gene Ontology (GO) Analysis

GO analysis is a commonly applied method for functional studies of large-scale genomic or transcriptomic data [12]. The Gene Ontology Enrichment Analysis Software Toolkit (GOEAST, http://omicslab.genetics.ac.cn/GOEAST/php/illumina.php) was used to identify significantly enriched GO terms among the given list of genes that are differentially expressed in response to IR. Statistically overrepresented GO categories with p-value <0.01 were considered significant.

### Ingenuity Pathway Analysis (IPA)

IPA software (Ingenuity System, Redwood City, CA) was employed to analyze our RNA-seq data in terms of biological responses and canonical pathways. Ranking and significance of the biofunctions and the canonical pathways were tested by the p-value. Additionally, canonical pathways were ordered by the ratio (number of genes from the input data set that map to the pathway divided by the total number of molecules that exist in the canonical pathway). IPA also generated cellular networks where the differentially regulated genes can be related according to previously known associations between genes or proteins, but independently of established canonical pathways. Top networks represented associative network functions based on a score that considers the –log (*p*-value), which aggregates the likelihood of the genes in the network being found together due to random chance. Score ≥2 was considered significant.

### Real-time Reverse Transcription (RT)-PCR

Total RNA was subjected to RT with random hexamers using the SuperScript First-Strand Synthesis System (Invitrogen, Carlsbad, CA) to obtain cDNAs. Real-time PCR was performed using a Power SYBR Green PCR Master Mix (Applied Biosystems, Foster City, CA) according to the manufacturer’s instructions. Threshold cycles (Ct) were automatically calculated by the Mastercycler ep realplex (Eppendorf, Hamburg, Germany). PCR condition was as follows: polymerase activation at 95°C for 10 min, followed by 40 cycles of 95°C for 15 sec, 60°C for 1 min. The intensity of each gene was normalized against GAPDH expression. Differential expression was calculated as a ratio of the expression levels of target genes in irradiated and non-irradiated cells, according to the ΔΔ C*t* method [13]. For all primers pairs, specific amplification of the PCR products was confirmed by melting curve analysis and agarose gel electrophoresis. Primers were designed using Primer Express® 4.0 (Applied Biosystems, Foster, CA) and the sequences are presented in Table 1.

**Table 1:** Primer pairs of real-time RT-PCR analysis.

Gene	Gene name	Primers from 5′ to 3′	Size (bp)
SESN2	Sestrin 2	F: ACTGCGTCTTTGGCATCAG	101
		R: GTAGCAGGCCACTGTCTTGA	
FN1	Fibronectin 1	F: GGAGTTGATTATACCATCACTG	259
		R: TTTCTGTTTGATCTGGACCT	
TRAF4	Tumor necrosis factor (TNF)	F: GCTGCATCCACAGTGAGGAGGG	317
	receptor-associated factor 4	R: CTCAGAGGTGGCATGCTGGGCC	
CDKN1A	Cyclin-dependent kinase inhibitor 1, p21	F: GGACCTGGAGACTCTCA	90
		R: CCTCTTGGAGAAGATCAG	
COX-2	Cyclooxygenase 2,	F: TTCAAATGAGATTGTGGGAAAAT	305
	Prostaglandin-endoperoxide synthase 2 (PTGS2)	R: AGATCATCTCTGCCTGAGTATCTT	
DDB2	DNA damage binding protein 2	F: CCCTGAACCCATGCTGTGAT	132
		R: TCGGGACTGAAACAAGCTGC	
FDXR	Ferredoxin reductase	F: GGAAATTCCTGGTGAGGAGC	366
		R: CTGGAGACCCAAGAAATCCAC	
GAPDH	Glyceraldehyde-3-phosphate	F: ATGACATCAAGAAGGTGGTG	177
	dehydrogenase	R: CATACCAGGAAATGAGCTTG	

### Western Blot Analysis

After 4 hr irradiation, total cell lysates (5×10^6^ cells) were prepared using RIPA lysis buffer (50 mM Tris, pH 7.4, 150 mM NaCl, 1% NP-40, 1 mM NaF, 1 mM EDTA, 1 mM Na_3_VO_4_, 1 mM PMSF, 0.25% Na-deoxycholate, and 5 U/ml aprotinin). For western blot analysis, denaturated protein lysates (40 µg) were subjected to SDS-PAGE. Separated pro­teins were transferred to a nitrocellulose membrane and blocked with 5% skim milk in TBST (10 mM Tris, 100 mM NaCl, and 0.1% Tween 20) for 1 hr at room temperature. The membranes were then probed with specific primary antibodies followed by peroxidase-conjugated secondary antibody, and visualized using an ECL detection system (GE Healthcare, Little Chalfont, Buckinghamshire, England).

## Results

### Design of RNA-seq Study in Radioresistant NSCLC Cells

**Figure 1:**
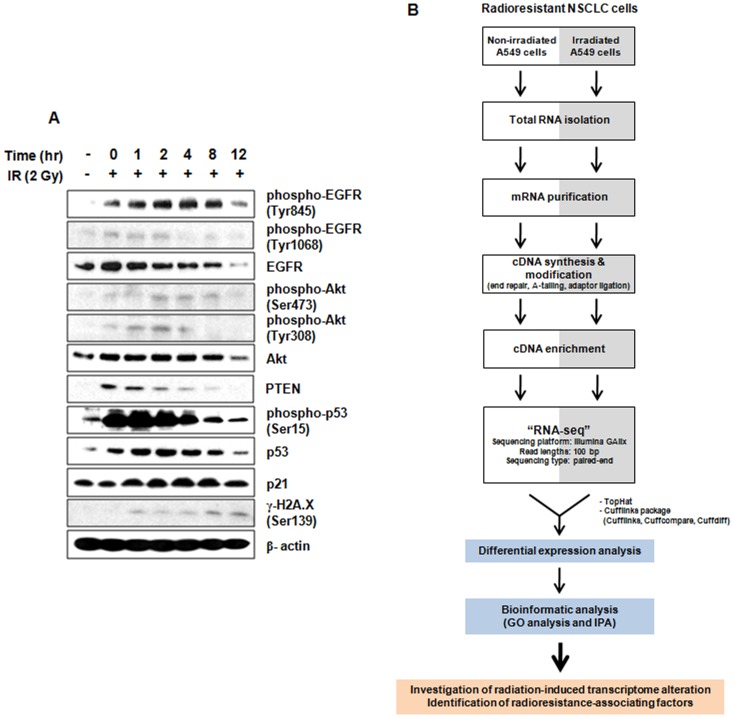
Design of RNA-seq study in radioresistant NSCLC A549 cells. (A) Determination of appropriate irradiation condition based on expression of representative radioresponsive proteins. (B) A schematic diagram for design and goals of our study. TopHat aligns RNA-seq reads to genome reference (hg19) and finds transcript splice sites. Cufflinks assemble the reads generated from TopHat alignment into transcripts. Cufflinks package consists of the following software - Cufflinks, assembles transcrips; Cuffcompare, compares transcript assemblies to annotation; Cuffdiff, finds differentially expressed genes and transcripts.

Each cell line derived from NSCLCs has been reported for their different radiosensitivity. A549 cells, a well-characterized radioresistant NSCLC cell line, show significant tolerance to radiation and modest loss of cell viability after exposure to IR [4], [14]. In this study, we would like to investigate radiation-induced whole transcriptome alteration in radioresistant NSCLC cells using RNA-seq, and furthermore to identify radioresistance-associating factors (potential biomarkers of radioresistance). In order to verify the appropriate experimental conditions for the RNA-seq analysis, we examined the time-dependent expression of several proteins known as radioresponsive factors [15], [16], [17] in radioresistant NSCLC A549 cells under irradiation. Also, considering cumulative effects of radiation, irradiation dose was set at 2 Gy. This was within the dose range typically administered in radiation biology experiments involving cells [18]. As shown in Fig. 1A, the expression levels of phospho-EGFR (Tyr845), phospho-Akt (Ser473) and p21 showed a maximum increase at 4 hr after irradiation. Additionally, IR-induced PTEN expression was gradually decreased, while phosphoryaltion of Akt at Ser473 was increased in a time-dependent manner.

Upon the purpose of experiment, sequencing platforms, read lengths and sequencing types should be properly determined. These experimental conditions are closely related to efficiency of RNA-seq analysis. Illumina sequencing platform appears to be highly replicable with relatively little technical variation such as transcript-length bias [19]. It shows greater transcriptome coverage and sequencing depth [10]. Moreover, paired-end sequencing is suitable for conducting qualitative analysis such as transcription start site mapping, detection of gene fusion transcripts and alternative splicing, and mapping of genomic structural variations including deletions, insertions and rearrangements [20]. Based on these research results, after 4 hr irradiation, total RNA was isolated from irradiated and non-irradiated radioresistant A549 cells, used to create Illumina RNA-seq library, and then subjected to 100 bp paired-end sequencing using Illumina GAIIx platform. A schematic diagram for the design and goals of our study is presented in Fig. 1B.

### Identification of Radioresponsive Genes in Radioresistant NSCLC Cells through Transcriptome Analysis

Total numbers of RNA-seq reads (result format: FASTQ) acquired from irradiated and non-irradiated radioresistant NSCLC A549 cells, were 32,315,026 (6,527,635,252 bp) and 31,084,922 (6,279,154,244 bp), respectively. We aligned the sequence reads to a human genome reference (hg19) using TopHat version 1.2.0. Splice junctions were extracted from RefSeq alignment (downloaded from the University of California, Santa Cruz (UCSC) Genome Browser). In total, at least one ends of 28,372,827 (87.8%) and 27,514,052 (88.5%) reads for the irradiated and non-irradiated A549 cells, respectively, were successfully mapped against hg19. Next, the resulting read alignments (file format: BAM) were assembled through Cufflinks version 1.0.3, and it created a novel transcript using Cufflinks reference annotation-based transcript (RABT) algorithm. The transcripts combined by Cuffcompare were used to calculate relative abundances of each transcript through Cuffdiff. Gene expression levels were determined by measuring the sum of fragments per kilobase of exon model per million mapped reads (FPKM) values of its exons. To acquire more accurate results, we filtered out the each data, if estimated FPKM values in both irradiated and non-irradiated sample were less than 1.0 (cf. FPKM value of 0.05 as the lower bound of expression level is commonly set). Ultimately, we identified that 727 genes were significant differentially expressed in radioresistant A549 cells after irradiation. Among these genes, 367 genes were up-regulated, and 360 genes were down-regulated. According to cellular functional categorization, significantly up-regulated genes were classified as follows: cell cycle (*CDKN1A, MLL5, NEK11, TP53INP1*), repair (*DDB2, REV3L, SESN1, SESN2, XPC*), cell death (*ACER2, BTG2, MDM2, MEF2A, OPA1, PLTP, TRIAP1*), lipid metabolism (*COL4A3, COX-2*), cell growth and proliferation (*DOK1, EIF2AK2, FDXR, DFRK, GDF15, GSTM1, PDK1, PGF, PIK3IP1, PPM1D, RHOBTB2, SH3BP2, SHBG, SLC22A1*) and immune system responses (*FOXP3, TNFSF9*). Also, significantly down-regulated genes were classified as follows: cell cycle (*CDC42, MT2A, SPC25, STAT5A*), repair (*H2AFX, PDE11A, XRCC3*), cellular development (*GPER, ICAM2, OPHN1*), cell death (*CARD8, CEACAM1, PTPRG, ST6GAL1*), cell growth and proliferation (*BAI1, F3, KLF11, MNT, MORF4L1, MYC, ROMO1, SMAD1, ST7L, TBXA2R*) and immune system responses (*IL12A*). Detailed results are shown in Dataset S1, Table S1 and Table S2.

**Figure 2:**
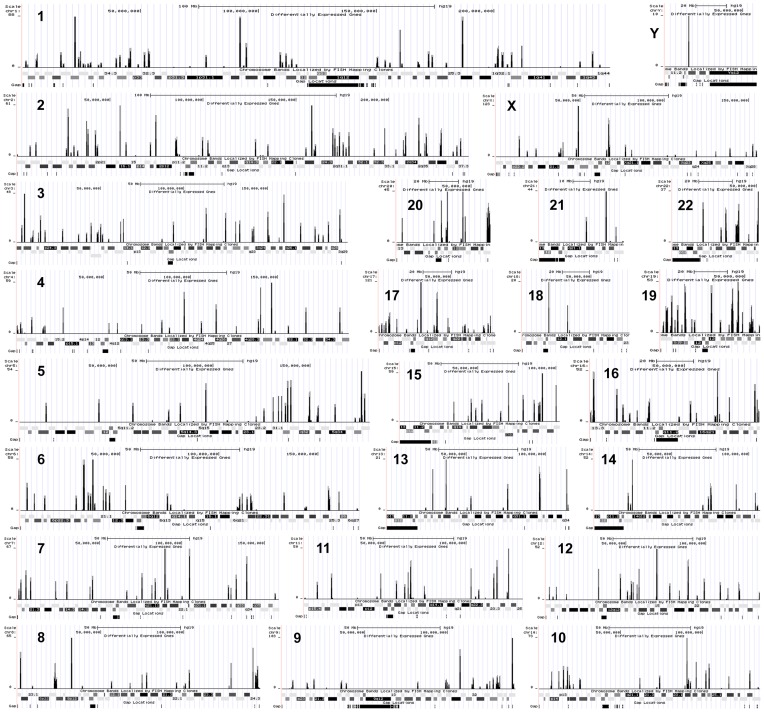
Chromosome distribution patterns of differentially expressed genes of radioresistant A549 cells in response to IR. (x-axis: chromosome coordinate, y-axis: the number of differentially expressed genes of A549 cells under irradiation in 400 kb sliding window).

Furthermore, to verify the characteristic chromosomal locations of genes controlling radiation responses, we examined the expression landscape across whole chromosomes by investigating alteration of gene expression in irradiated radioresistant A549 cells. In 400 kb sliding window, the number of differentially expressed genes of A549 cells in response to IR, is plotted along with the whole chromosomes using custom-track of UCSC Genome Browser (Fig. 2). We found that chromosome distribution patterns varied greatly with respect to gene density, and especially chromosome 19 showed the highest gene density of mapped genes. In radioresistant NSCLC A549 cells, a large number of genes showing IR-altered expression were located on chromosome 1, 2, 3 and 19. However, we could not obtain meaningful information of direct relationship between radiation responses and these chromosome distribution patterns.

### Investigation of Radiation-induced Transcriptome Alteration in Radioresistant NSCLC Cells through GO Analysis

**Figure 3:**
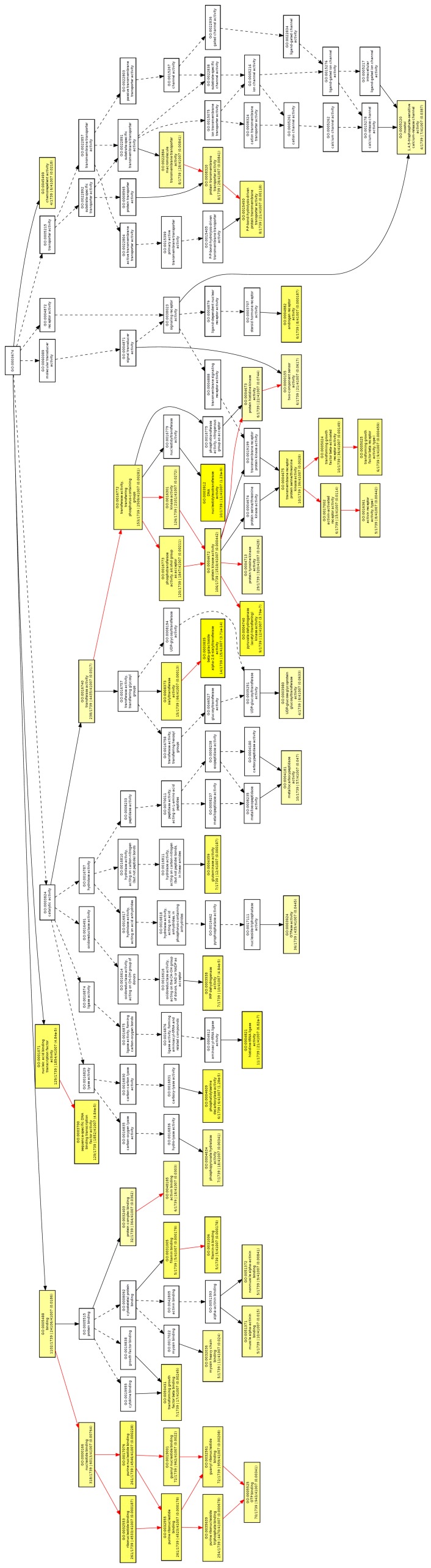
GOEAST graphical output of enriched GO terms in molecular function ontology for IR-induced transcripts from radioresistant A549 cells. Each box has GO terms labeled by its GO ID, term definition and detailed information representing ‘q/m|t/k (p-value)’. *q* is the number of genes associated with the listed GO ID (directly or indirectly) in our data set, *m* is the number of genes associated with the listed GO ID (directly or indirectly) on the selected platform, *k* is the total number of genes in our data set, *t* is the total number of genes on the selected platform, and p*-*value represent significance of the enrichment in the data set of the listed GO ID with hypergeometric distribution. Branches of the GO hierarchical tree without significantly enriched GO terms are not presented. The degree of color saturation of each box is positively associated with the enrichment significance of the corresponding GO term. Significantly enriched GO terms are indicated in yellow boxes. Insignificant GO terms within the hierarchical tree are shown as white boxes. Arrows show correlations between different GO terms. Red arrows reveal relationships between two enriched GO terms, black solid arrows reveal relationships between enriched and unenriched terms, and black dashed arrows reveal relationships between two unenriched GO terms.

Within any given gene sets (microarray or NGS experimental data sets), GO-based functional analysis provides statistically enriched GO terms, which describe gene products and demonstrate their relationships according to three ontology categories: biological process, molecular function and cellular component [12]. To analyze our RNA-seq data based on groups of functionally related genes instead of individual genes, we used GOEAST as a web-based high-throughput functional genomic analysis tool. We then identified significantly enriched GO terms and characterized radiation responses of radioresistant NSCLC A549 cells. In total, enriched GO terms were found in 77 subcategories under biological process, 17 subcategories under cellular component, and 50 subcategories under molecular function. In biological process ontology, the results indicated that nuclear localization, TGF-β receptor signaling pathway, cell cycle arrest, cell migration, serine/threonine kinase signaling pathway, angiogenesis, BMP signaling pathway, and regulation of cell morphogenesis are mainly associated with radioresponses in radioresistant A549 cells. Focal adhesion was one of the major cellular components modified by IR. Significant GO enrichment profiles in the biological process and cellular component categories (only p-value less than 0.01) are summarized in Table 2. We also determined the overrepresented GO terms in a graphical format according to their relationships in the hierarchical tree of molecular function ontology (Fig. 3). Enriched GO molecular function terms in radioresistant A549 cells under irradiation were β-galactoside α-2,6-sialyltransferase activity, pyruvate dehydrogenase kinase activity, TGF-β receptor activity, GTP binding, protein transmembrane transporter activity, filamin binding and activin receptor activity. Through GO analysis, we found that overrepresented GO terms in our radioresponsive gene sets, are closely linked to EMT, migration and further angiogenesis. With involvement of focal adhesion components, TGFβ/BMP signaling, filamin binding and activin receptor activity have been reported to regulate alteration of cell morphology during EMT or cell migration. Taken together, we suggest that EMT and EMT-related events may be critical in regulating radiation responses in radioresistant A549 cells under irradiation.

**Table 2:** GO category enrichment profile of radioresistant A549 cells in response to IR (p-value<0.01).

GO ID		Enriched significant GO category	p-value		Significant gene symbols
**Biological process**					
GO:0007172	Signal complex assembly		4.20E−08	MAPK8IP2, PTK2	
GO:0051647	Nucleus localization		4.65E−08	CDC42, PTK2	
GO:0006427	Histidyl-tRNA aminoacylation		6.82E−07	HARS2	
GO:0007179	Transforming growth factor beta (TGF-β) receptor signaling pathway		1.69E−06	ACVR1, FURIN, GDF15, ID1, SMAD1, TGFBR3	
GO:0071157	Negative regulation of cell cycle arrest		6.31E−06	MDM2	
GO:0043542	Endothelial cell migration		8.74E−06	CYP1B1, ID1, HSPB1, PTK2	
GO:0007178	Transmembrane receptor protein serine/threonine kinase signaling pathway		7.25E−05	ACVR1, BMPR1B, FURIN, GDF15, ID1, SMAD1, TGFBR3	
GO:0055108	Golgi to transport vesicle transport		0.0001783	EXOC4	
GO:0046620	Regulation of growth		0.0001783	NRG1, PTK2, SERP1	
GO:0051640	Organelle localization		0.0005195	CDC42, COPA, GPSM2, MEF2A, MLPH, MYO5A PTK2, SAR1B	
GO:0001525	Angiogenesis		0.0006979	ACVR1, CEACAM1, CYP1B1, FN1, HSPB1, HSPG2, ID1, NAA15, PGF, SLC12A6	
GO:0010721	Negative regulation of cell development		0.0012465	ACVR1, CEACAM1, CYP1B1, FN1, HSPB1, HSPG2, ID1, NAA15, PGF, SLC12A6	
GO:0030509	Bone morphogenic protein (BMP) signaling pathway		0.0028114	ACVR1, BMPR1B, ID1, SMAD1, SMAD5, TGFBR3	
GO:0030174	Regulation of DNA-dependent DNA replication initiation		0.0044184	CDT1, CIZ1	
GO:0000226	Microtubule cytoskeleton organization		0.0046760	BLOC1S2, CCDC88B, CCDC88C, CEP250, CHD3, GPSM2, HAUS7, KIF23, MARK4, OFD1, PRC1, PTK2, SPC25	
GO:0022604	Regulation of cell morphogenesis		0.0051651	CDC42EP1, CDKL5, FN1, LARP4, NOTCH1, PLXNB2, PTK2, RUFY3, SEMA3A, SEMA3F, SMAD1, XYLT1	
GO:0031344	Regulation of cell projection organization		0.0070184	CDC42, CDC42EP1, CDKL5PLXNB2, PTK2, RUFY3, SEMA3A, SEMA3F, SMAD1, XYLT1	
GO:0007021	Tubulin complex assembly		0.0076473	TBCA	
GO:0018106	Peptidyl-histidine phosphorylation		0.0084104	PDK1, PDK2	
GO:0030070	Insulin processing		0.0086510	CPE	
**Cellular component**					
GO:0045254	Pyruvate dehydrogenase complex		8.35E−05	PDK1, PDK2	
GO:0005925	Focal adhesion(Cell-substrate adherens junction)		0.0010309	PDLIM2, PTK2, TENC1	
GO:0048179	Activin receptor complex		0.0021545	ACVR1	
GO:0000015	Phosphopyruvate hydratase complex		0.0034055	ENO3	

### Investigation of Radiation-induced Transcriptome Alteration in Radioresistant NSCLC Cells through IPA Analysis

**Figure 4:**
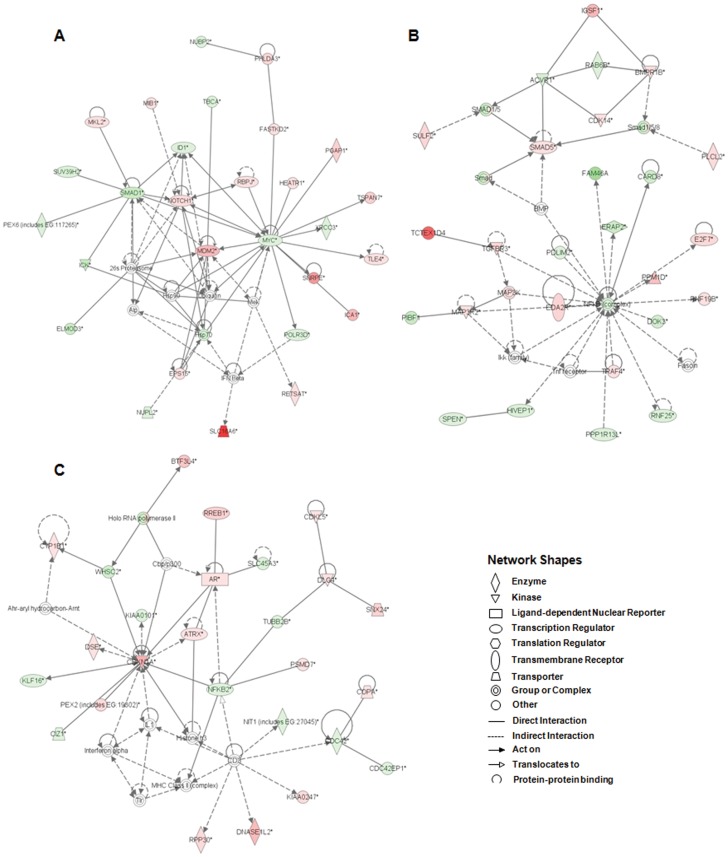
The top three ranked networks identified by IPA from the entire transcripts of IR-induced radioresistant A549 cells. (A) Network for cell death, post-translational modification, and protein folding (score: 38). (B) Network for cellular development, connective tissue development and function, and cancer (score: 37). (C) Network for cell cycle, cellular development, and hematological system development and function (score: 37). Networks are displayed graphically as nodes (genes/gene products) and edges (biological relationships between the nodes). Intensity of the node color indicates the degree of regulation (red: up-regulation, green: down-regulation, white: not differentially expressed but related to this network).

IPA was performed for functional analysis of our radioresponsive gene sets acquired from the RNA-seq. As a result, we verified 25 networks, top biofunctions (22 diseases and disorders and 27 molecular and cellular functions) and 177 canonical pathways concerned with the radiation-induced transcriptome alteration in radioresistant A549 cells. The most significantly related biofunctions and canonical pathways (only p-value less than 0.01) are shown in Table 3. Based on the results in IPA biofunctions under diseases and disorders category, we first proposed that immunological/inflammatory responses could be meaningfully associated with responses to IR in radioresistant A549 cells. In addition, among 25 networks, top seven networks supplemented with IPA-score, number of focus genes, biofunctions, and hub genes/hub-interacting partners are presented in Table 4. The high-scoring functions in the networks were cell death, connective tissue development and function, cell cycle, lipid metabolism, DNA repair, cell death, cell morphology and immune cell trafficking. The top three scoring networks, which could be considerably responsible for radioresponses in radioreisistant A549 cells, are illustrated in Fig. 4. The detailed cellular functions of each network were as follows: (i) cell death, post-translational modification, and protein folding (IPA-score of 38), (ii) cancer, cellular development, and connective tissue development and function (IPA-score of 37), and (iii) cell cycle, cellular development, and hematological system development and function (IPA-score of 37). 20 hub genes as key regulators of radiation responses in radioresistant A549 cells, were presented in Table 4. They were as follows: *AR, BID, CAMK2D, CDC42, CDKN1A, DLG4, EIF2AK2, IFIT3, MEF2A, MDM2, MYC, MYOCD, NFKB2, NOTCH1, COX-2, PTK2, SMAD1, SMAD5, TRAF1* and *XPC*. Most hub genes in the networks are mainly associated with cell cycle, cell proliferation and apoptosis. However, with immunological/inflammatory responses in biofunction category earlier mentioned, it is worth of notice that COX-2 playing a critical regulatory role in inflammation was detected as a hub gene involved in radiation responses of radioresistant NSCLC A549 cells.

**Table 3:** Top-associated biofunctions and canonical pathways by IPA.

Biofunctions - Diseases and disorders	p-value (<0.01)		#Molecules
Cancer	9.08E−04–2.32E−02		193
Gastrointestinal disease	9.08E−04–2.35E−02		209
Genetic disorder	1.15E−03–2.35E−02		107
Immunological disease	3.37E−03–2.15E−02		21
Inflammatory response	5.67E−03–1.58E−02		14
Connective tissue disorder	6.58E−03–8.6E−03		7
Inflammatory disease	1.07E−02–2.35E−02		65
**Biofunctions - Molecular and cellular functions**			
Cell cycle	1.79E−06–2.52E−02		71
Cellular assembly and organization	4.48E−05–2.51E−02		90
Cell-To-Cell signaling and interaction	7.09E−05–2.51E−02		45
DNA replication, recombination and repair	1.51E−04–1.85E−02		65
Cell death	3.03E−04–2.51E−02		109
Cellular development	3.45E−04–2.45E−02		89
Cell morphology	3.83E−04–2.25E−02		58
Molecular transport	5.21E−04–2.46E−02		42
Cellular movement	7.18E−04–2.51E−02		51
Cellular growth and proliferation	7.77E−04–2.45E−02		173
**Canonical pathways**	**-log(p-value)**	**Ratio**	**#Molecules**
Small cell lung cancer signaling	3.20E+00	1.01E−01	9
p53 signaling	2.92E+00	1.04E−01	10
EIF2 signaling	2.84E+00	7.54E−02	15
Molecular mechanisms of cancer	2.53E+00	5.80E−02	22
Aryl hydrocarbon receptor signaling	2.16E+00	6.92E−02	11

**Table 4:** IPA network analysis of radioresponsive genes in radioresistant A549 cells.

Score^a^	Number of focus genes	Top functions^b^	Hub genes^c^	Interacting partners^d^
38	28	Cell death, Post-translational	MDM2	TBCA, SMAD1
		modification, Protein folding	MYC	FASTKD2, HEATR1, ICA1, PGAP1, POLR3D, RBPJ, SNRPE, TLE4, XRCC
			NOTCH1	ID1, MYC, RBPJ, SMAD1
			SMAD1	ICK, ID1, MDM2, MKL2, NOTCH1, SUV39H2
37	26	Cancer, Cellular development, Connective tissue development and function	NFKB2	CARD8, DOK3, E2F7, EDA2R, ERAP2, FAM46A, HIVEP1, MAP3K, PDLIM2,PPM1D, RNF19B, RNF25, TGFBR3, TRAF4
			SMAD5	ACVR1
37	26	Cell cycle, Cellular development, Hematological	AR	ATRX, NFKB2, RREB1, SLC45A3
		system development and	CDC42	CDC42EP1, COPA
		function	CDKN1A	AR, ATRX, CIZ1, DSE, KIAA0101, KLf16, NFKB2, PEX2, WHSC2
			DLG4	CDKL5, SNX24, TUBB2B
			NFKB2	AR, CDKN1A, PSMD7, TUBB2B
35	25	Reproductive system	CAMK2D	ACTB, MYO5A
		development and function, Drug metabolism, Endocrine system development and function	IFIT3	MAVS
21	20	Lipid metabolism, Small molecule biochemistry, Cellular	MEF2A	ENO3, MEF2A, MYOCD, SLC2A4RG
		development	MYOCD	MEF2A, ZNF354A
			COX-2	AGER, IGFBP7, MEF2A, TRAF1, VCAN
			TRAF1	COX-2, ZMAT3
19	20	Repair, Cell death, DNA	BID	FAS, H2AFX, HSPA1A/1B
		replication, Recombination, Cell	PTK2	FAS
		morphology	EIF2AK2	DNAJC3, HSPA1A/1B
17	16	Cell-To-Cell signaling and interaction, Hematological system development and function, Immune cell trafficking	XPC	DDB2, KIN

a The score provides the networks a measure of how accurate the focus genes are matched. The assessment is based on the number of focus genes and network size. For details, refer to the following web site (Ingenuity Systems Pathway Analysis, http://www.ingenuity.com).

b The assignment of functions to a network is based in literature stored in the IPA Knowledge Base.

c Hub genes were selected in the basis of at least three interactions with differently expressed genes.

d Interacting partners are the genes that served for identification of respective Hub genes.

### Real-time RT-PCR Validation of the RNA-seq Results

**Figure 5:**
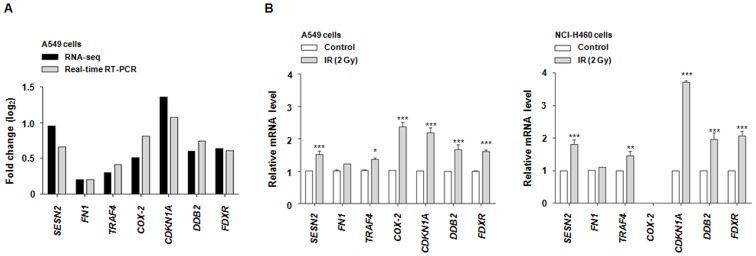
Accurate validation of our RNA-seq data. (A) Correlation of differential expression between RNA-seq and real-time RT-PCR. The log_2_ ratios were generated by comparing expression levels in irradiated to non-irradiated radioresistant A549 cells. (B) Suggestion of gene expression-based putative biomarkers of radioresistance in NSCLC cells. Amplification of the GAPDH fragment in PCR was used as control. The results were confirmed by three independent experiments. Data was presented as mean ± standard deviation (SD) and analyzed using the one-way ANOVA on ranked data, followed by a Tukey's honestly significant difference test and the two-way ANOVA on ranked data, followed by a Bonferroni post test using Prism 4 (GraphPad Software, San Diego, CA). *p-value <0.05; irradiated cells vs. control cells, **p-value <0.01; irradiated cells vs. control cells, ***p-value <0.001; irradiated cells vs. control cells.

Through GO analysis and IPA, we found that EMT-associated events, cell migration and inflammatory process are closely related to radiation responses in radioresistant NSCLC A549 cells. We further investigated the relationship between these radioresponses and the hub genes/hub-interacting genes in IPA network analysis. Based on the results, we selected seven possible candidate genes for significant radiation-altered factors (*SESN2, FN1, TRAF4, CDKN1A, COX-2, DDB2* and *FDXR*). To validate the precision of our RNA-seq data, real-time RT-PCR was performed on these selected seven genes. It was carried out under the same conditions as those used for the RNA-seq analysis. A comparison of fold changes between RNA-Seq and real-time RT-PCR for each gene (*SESN2, FN1, TRAF4, CDKN1A, COX-2, DDB2* and *FDXR*) is shown in Fig. 5A. As a result, we verified that the real-time RT-PCR expression profiles were in almost complete agreement with the RNA-seq data. Although there were small differences in the fold change values between two methods of measurement, these results were generally highly related, strongly supporting the reliability of our RNA-seq analysis.

### Identification of Candidates for Radioresistance-associating Factors in NSCLC Cells

**Figure 6:**
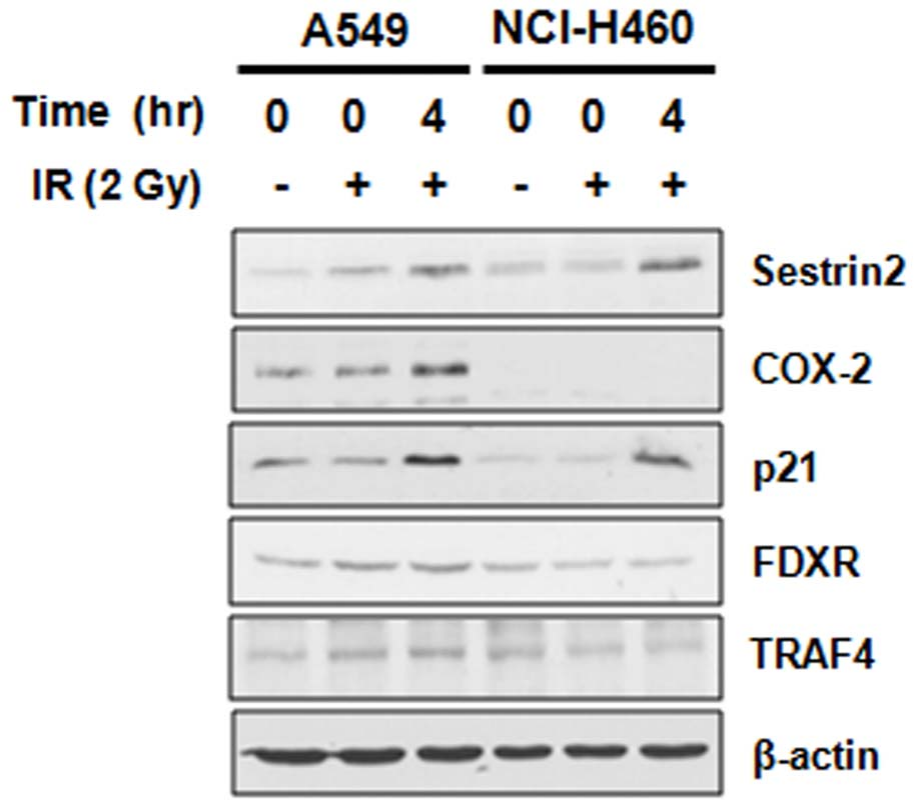
Protein expression of candidate genes for radioresistance-associating factors in NSCLC cells. IR-altered expression of Sestrin2, TRAF4, p21, COX-2 and FDXR was assayed by western blot analysis. The results were confirmed by three independent experiments.

A549 and NCI-H460 cells can be used as models for radioresistant and radiosensitive NSCLC cells, respectively, due to their considerable differences in radiosensitivity [4], [14]. To verify whether or not *SESN2, FN1, TRAF4, CDKN1A, COX-2, DDB2* and *FDXR* could be related to cellular radioresistance in NSCLC cells, the mRNA expression of these genes was compared between radioresistant A549 and radiosensitive NCI-H460 cells through real-time RT-PCR (Fig. 5B). The results indicated that *COX-2* and *CDKN1A* show significantly different expression patterns in both cell lines in response to IR. Under irradiation, the most differentially expressed gene between A549 and NCI-H460 cells was *COX-2*. In radiosensitive NCI-H460 cells, *COX-2* was not detected even after irradiation. In 50 cycle real-time PCR condition, IR induced only modest expression of *COX-2*. Furthermore, except for *FN1* showing non-statistically significant fold change in both cell lines under irradiation (Fig. 5B), *SESN2, TRAF4, CDKN1A, COX-2* and *FDXR* as potential candidate genes for radioresistance-associating factors, were verified their protein expression levels in A549 and NCI-H460 cells. As shown in Fig. 6, IR increased protein expression of Setrin2, COX-2 and p21 in accordance with the results of RNA-seq and real-time RT-PCR. However, unlike IR-increased *FDXR* expression in both cell lines, FDXR protein was expressed at low levels irrelevant to IR irradiation in NCI-H460 cells. COX-2 revealed the most significant difference in IR-induced protein expression between radioresistant A549 and radiosensitive NCI-H460 cells. IR-induced increase of COX-2 expression was appeared only in radioresistant A549 cells. Based on differential gene expression of COX-2 in these two types of NSCLC cells, we suggest that COX-2 could have possibility as a biomarker of radioresistance in NSCLC cells.

## Discussion

Lung cancer is the most frequently diagnosed cancer type as well as the leading cause of cancer death [21]. Although considerable progresses have been made in early diagnosis and treatment of lung cancer, the recurrence rates remain high. Lung cancer is often diagnosed at the inoperable advanced stages [22]. Radiotherapy is a common treatment strategy applied for the local tumor control, however unfortunately the therapeutic outcomes are not fully satisfactory. Radioresistance is considered a main impediment to successful radiation treatment of a variety of malignancies including lung cancer. Until now, cause and mechanism of radioresistance during and after irradiation are still largely obscure. Several oncogenes such as Myc, Ras and Pim-1 have been reported for the radioresistance [4], [23], [24], but understanding the mechanism of a single gene/protein-linked radioresistance is not entirely accurate. Besides, some reports are occasionally contradictory. Therefore, global approaches are demanded to obtain a comprehensive view of radioresistance mechanism.

In this study, we aimed to examine the extensive radioresponses at entire transcriptome level in radioresistant NSCLC cells and to identify radioresistance-associating factors. Using RNA-seq, we investigated IR-induced whole transcriptome alteration in irradiated and non-irradiated NSCLC A549 cells. Comparing the transcriptome profiles in response to IR, we identified 727 genes with significant differential expression between irradiated and non-irradiated A549 cell. Also, we examined the expression landscape across whole chromosomes by investigating alteration in radiation-induced gene expression. We found that a large number of genes showing IR-altered expression were located on chromosome 1, 2, 3 and 19, and especially chormomosome 19 revealed the highest gene density. However, we could not obtain meaningful information about direct relevance between these characteristic chromosome distribution and radiation responses. Next, functional analysis of the radioresponsive genes was performed by bioinformatic approaches (GO analysis and IPA). We found that EMT, cell migration and inflammatory processes could be meaningfully related to regulation of radioresponses in radioresistant A549 cells. Based on the results of bioinformatic analysis, we selected seven significant radiation-altered genes (*SESN2, FN1, TRAF4, CDKN1A, COX-2, DDB2* and *FDXR*). To investigate the association between these radioreponsive genes and radioresistance, we compared the expression of these seven genes in radioresistant A549 cells and radiosensitive NCI-H460 cells. Taken together, we proposed a putative critical radioresistance-associating factor, COX-2.

Transcriptome is a complete set of all RNA molecules including mRNAs, non-coding RNAs and other small RNAs in a cell. Because gene expression is a major determinant of cellular phenotypes and functions, quantification of the expression levels of each transcript and determination of the transcriptional structure of genes are required to understand cellular responses during development and under specific conditions such as disease. For these reasons, several methods for transcriptome research have been established.

RNA-seq is the first sequencing-based approach that allows quantitative measurement of transcriptome in a high-throughput manner. It has several advantages to overcome the limitations of existing methods. First, RNA-seq is not restricted to detection of transcripts that correspond to existing genomic sequences. Second, RNA-seq has a low experimental background signal due to DNA sequences definitely mapped to unique regions of the genome. Third, RNA-seq does not reveal an upper restriction for quantification, which directly associates with the number of acquired sequences. Therefore, it can possess a high dynamic range of expression levels over which transcripts can be detected. Fourth, RNA-seq represents a high accuracy for quantifying expression levels, as determined using real-time RT-PCR. The results of RNA-seq also show high levels of reproducibility, for both technical and biological replicates [11]. As shown in Fig. 5A, we verified that correlation between real-time RT-PCR fold change and RNA-seq fold change was significant, strongly supporting the reliability of our RNA-seq analysis. Finally, since there are no cloning steps, RNA-seq requires only a small amount of RNA sample, and it is even possible to work at single cell resolution [25]. Another important advantage of RNA-seq is an ability to discover unknown transcripts. Paired-end sequencing, used in our study, provides elevated physical coverage and alleviates several restrictions of NGS platforms that arise because of their relatively short read length. It enables us to investigate alternative splicing patterns without demand for knowledge of transcript annotations, and also has a merit of gene fusion identification due to the increased physical coverage it offers [26]. In this study, we would like to examine the qualitative regulation of gene expression in radioresistant A549 cells under irradiation. For these purposes, we accomplished paired-end sequencing using Illumina GAIIx platform. However, there were not significant and meaningful radiation-induced alterations in alternative splicing and gene fusion event (data not shown). Further study focusing on a qualitative analysis, is needed to understand the exact radioresistance mechanism.

Differential gene expression profiles from multiple experimental conditions have been reported. Next, we compared the results derived by our RNA-seq analysis to the existing microarray databases of A549 cells irradiated IR [27], [28]. In both RNA-seq and cDNA microarray data, a number of genes involved in regulation of cell cycle, DNA repair, apoptosis and cell proliferation was responsible for the significant responses to IR in radioresistant A549 cells, and also TP53 and MDM2 were the most significant radiation-altered genes. Beyond these genes, induction of well-known radioresponsive genes (*CDKN1A, DDB2, MYC, XPC* and *XRCC*) are detected in both RNA-seq and microarray data. However, only in our RNA-seq results combined with bioinformatic analysis, inflammatory process is distinctively suggested as a critical radioresponse in radioresistant NSCLC A549 cells. Until now, the precise mechanisms of association of inflammation with radiation-induced damages such as radioresistance have not yet been established. Another recent microarray study reported that human solute carrier (SLC) gene superfamily were overexpressed in radioresistant A549 cells, and increased expression of SLC genes might contribute to radioresistant phenotype [29]. In our study, we also found that a large number of SLC genes (SLC7A, SLC12A, SLC22A, SLC25A, SLC27A, SLC30A superfamily, SLC4A5, SLC16A6, SLC23A1, SLC31A2, SLC35E3, SLC35F2, SLC37A1, SLC38A1, and SLC45A3) are altered in response to IR in radioresistant A549 cells. Some differences in terms of detailed subtypes and expression aspects (up-regulation and down-regulation) were revealed, however, in accordance with the previous study, SLC7A11 showed significant up-regulation in radioresistant A549 cells.

In complicated biological systems, molecular networks represent backbone of molecular activity within the cell. Recent studies have been emphasized interpretation of these networks under specific conditions such as disease, contrasting networks of different species and molecular types [30]. Because cellular networks cannot be explained by gene expression knowledge only, biological network analysis combined with biological sequence analysis, could provide useful information to elucidate cellular machinery and to predict protein function and interaction. In this research, gene expression data acquired from RNA-seq provided extensive knowledge of radiation-induced transcriptome alteration in radioresistant NSCLC cells (Dataset S1, Table S1 and Table S2), however, it was difficult to understand distinguishing aspects of radiation responses only in gene expression data itself. Thus, we expected that the RNA-seq results combined with bioinformatics approaches (GO analysis and IPA) are likely to improve the understanding radiation effects in radioresistant NSCLC cells and the identification of functionally relevant biomarkers for radioresponses such as radioresistance. To examine the characterization of radioresistant NSCLC A549 cells, we used GO enrichment programs. Our attempt to map the gene lists to the GO structure allowed us to identify particular GO categories that are provoked after irradiation in radioresistant A549 cells. In biological process ontology, the results indicated that nuclear localization, TGF-β receptor signaling pathway, cell cycle arrest, cell migration, serine/threonine kinase signaling pathway, angiogenesis, BMP signaling pathway and regulation of cell morphogenesis are deregulated in irradiated radioresistant A549 cells (Table 2). Also, enriched GO molecular function terms were β-galactoside α-2,6-sialyltransferase activity, pyruvate dehydrogenase kinase activity, TGF-β receptor activity, GTP binding, protein transmembrane transporter activity, filamin binding and activin receptor activity (Fig. 3). We found that ‘cell cycle’ was the most enriched GO category in the radioresistant A549 cells under irradiation. However, because deregulation of cell cycle is well-known response to radiation, it is not surprisingly. Instead, we focused on TGF-β/BMP signaling pathway, filamin binding property and activin receptor activity, indicating pivotal roles in initiation of EMT, migration and further angiogenesis [31], [32], [33]. In pathological situation such as tumorigenesis, EMT might endow cancer cells with enhanced motility and invasiveness. Indeed, oncogenic transformation is associated with signal transduction pathways promoting EMT [34]. Enhanced EMT has been reported to promote radioresistance *in vitro* and *in vivo* [35], [36]. In addition, the acquisition of cell motility is an important phenomenon of tumor cells with invasive and metastatic phenotypes. It is worthy of notice that focal adhesions modulating cell dissociation, were significantly detected in top-ranked cellular components in radioresistant A549 cells under irradiation (Table 2). Supporting these results, in molecular and cellular functions category of IPA, a number of genes involved in cell morphology and cellular movement were considerably correlated with radiation responses in radioresistant A549 cells (Table 3). In category of physiological system development and function, connective tissue development and function was ranked most importantly (data not shown). Taken together, we suggest that EMT-related events and cell migration might be critical in regulating radiation responses in radioresistant NSCLC A549.

Another network-based analysis program, IPA was applied to determine the transcriptional profiles and molecular networks of radioresistant A549 cells exposed to IR. We demonstrated that the functions of radioresponsive genes from the IPA library of canonical pathways are related to small cell lung cancer signaling, p53 signaling, EIF2 signaling, molecular mechanisms of cancer, and Aryl hydrocarbon receptor signaling (Table 3). As shown in Table 4, the high-scoring functions in the top-ranked networks were cell death, connective tissue development and function, cell cycle, lipid metabolism, DNA repair, cell death, cell morphology and immune cell trafficking. Gene networks controlling cell cycle, cellular development and cell death are critically associated with radiation responses in radioresistant A549 cell. These were coincident with the results reported by GO analysis. IPA network analysis also suggested that hub genes/hub-interacting partners in Table 4, could act as key regulators of cellular radioresponses. Among these regulators, TRAF family members revealed significant association with radioresponses in radioresistant A549 cells. Actually, we demonstrated that TRAF2 regulates radioresistance through direct interaction with ribosomal protein S3 in radioresistant NSCLC A549 cells in an IR-dependent manner [37]. In the results of IPA and GO analysis, DNA damage repair, apoptosis and cell cycle arrest are well-known radioresponses. As shown in Table 4, several radiation-associated genes, such as *CDKN1A, COX-2* and *DDB2*, have been reported to be related to above features, but others including *NOTCH1, AR, SESN2, FDXR* and *TRAF4* are novel candidates for radiation responses, especially radioresistance. These genes involve malignant characteristics of tumor progression including poor prognosis, metastasis and invasion. However, the accurate function of these novel genes involving radioresistance remains unclear, thus further investigation is needed.

Next, using real-time RT-PCR and western blot analysis, we compared the expression of seven radiation-altered genes (*SESN2, FN1, TRAF4, CDKN1A, COX-2, DDB2* and *FDXR*) in two NSCLC cells with different radiosensitivity, A549 and NCI-H460 (Fig. 5B and Fig. 6). Interestingly, under irradiation, *COX-2* showed the most significant difference in mRNA and protein expression between A549 and NCI-H460 cells. IR-induced increase of COX-2 expression was appeared only in radioresistant A549 cells. These data suggest that the overexpression of COX-2 mRNA and protein could be associated with radiation tolerance in NSCLC cells.

Recent studies elucidate that COX-2 plays a pivotal role in tumor growth and spread of established tumors by influencing mitogenesis, cellular adhesion, immune process, apoptosis, and metastasis. There are positive correlations between COX-2 overexpression and poor prognosis in patients with squamous cell carcinoma with uterine cervix who were treated with radiotherapy. Previous reports suggested that COX-2 expression may play a role for radioresistance through anti-apoptotic pathway [38]. The cells overexpressing COX-2 tend to be resistant to apoptosis by inducing Bcl-2 expression. Increased expression of COX-2 was observed in the tumor cells after irradiation, and COX-2 expression decreased in relation with Bcl-2 expression. However, elevated COX-2 expression in lung cancer has not yet been systematically compared with their radiation sensitivity. On the other hand, inflammation-associated toxic side effect has been reported as one of the clinical challenges in lung cancer treatment with radiotherapy [39]. Considering critical roles of COX-2 in inflammatory process [40], we supposed that overexpression of *COX-2* only in radioresistant A549 cells could be closely concerned with top-ranked IPA biofunction categories – immunological/inflammatory disease and inflammatory response. Based on our results and the biological property of COX-2 in pathological processes, collectively, we suggest that COX-2 is more likely to be a putative radioresisatance biomarker than radioresistance-associating factors. Furthermore, we suggest that COX-2 inhibitor may be attributable to enhance apoptosis induced by radiation, leading to radiosensitivity. We cannot fully explain the mechanism of radioresistance by COX-2, however, our data implicate that COX-2 might be possible therapeutic target for radioresistance in NSCLC. Further study is needed to confirm our conclusion.

In this study, for the first time, we investigated the radiation-induced transcriptome profile of radioresistant NSCLC A549 cells through RNA-seq and bioinformatic functional analysis. Comparison of the gene expression patterns between radioresistant A549 cells and radiosensitive NCI-H460 cells, we also suggested a possible biomarker for radioresistance, COX-2. Our research with the new methodological approach may provide a useful guideline for the experimental design of gene expression studies and for exploring novel routes to uncover the complete regulatory network involved in the radioresponses. Unfortunately, we used only two NSCLC cell lines, A549 and NCI-H460 cells, however it will be worth to investigate the differences of radiation responses in another tumor cell lines and lung tissue. Some of the identified novel targets (especially, among the hub genes/hub-interacting partners) could be potentially interested in further studies for radiation effects. Our research could help to interpret the complicated molecular mechanism for radioresponses such as radioresistance. Furthermore, it might contribute to identify another target gene for predictive biomarker improving radiotherapy.

## Supporting Information

Table S1 **Identification of significantly up-regulated genes in irradiated radioresistant A549 cells using RNA-seq.**
(DOC)

Table S2 **Identification of significantly down-regulated genes in irradiated radioresistant A549 cells using RNA-seq.**
(DOC)

Dataset S1 **This file contains the RNA-seq results used in this paper.**
(XLS)
