# Supplementary material for: Investigation of Radiation-induced Transcriptome Profile of Radioresistant Non-small Cell Lung Cancer A549 Cells Using RNA-seq
Source: PLoS One. 2013 Mar 22;8(3):e59319. doi: 10.1371/journal.pone.0059319 (PMC3606344; doi:10.1371/journal.pone.0059319)
Supplement: Table S1 — Identification of significantly up-regulated genes in irradiated radioresistant A549 cells using RNA-seq. (DOC) [file pone.0059319.s001.doc]

**Table S1**

Identification of significantly up-regulated genes in irradiated radioresistant A549 cells using RNA-seq

| **Gene** | **Gene name** | **Location** | **Type(s)** | **Entrez gene ID**  **for human** | **Ratio** |
| --- | --- | --- | --- | --- | --- |
| SLC22A1 | Solute carrier family 22 (organic cation transporter), member 1 | Plasma membrane | Transporter | 6580 | 2.348 |
| FOXP3 | Forkhead box P3 | Nucleus | Transcription regulator | 50943 | 2.285 |
| NRL | Neural retina leucine zipper | Nucleus | Transcription regulator | 4901 | 1.975 |
| GSTM1 | Glutathione S-transferase mu 1 | Cytoplasm | Enzyme | 2944 | 1.583 |
| RSPH1 | Radial spoke head 1 homolog (Chlamydomonas) | Nucleus | Other | 89765 | 1.549 |
| COL4A3 | Collagen, type IV, alpha 3 (Goodpasture antigen) | Extracellular space | Other | 1285 | 1.504 |
| SHBG | Sex hormone-binding globulin | Extracellular space | Other | 6462 | 1.504 |
| CMPK2 | Cytidine monophosphate (UMP-CMP) kinase 2, mitochondrial | Cytoplasm | Kinase | 129607 | 1.113 |
| SESN1 | Sestrin 1 | Nucleus | Other | 27244 | 1.090 |
| ALS2CR8 | Amyotrophic lateral sclerosis 2 (juvenile) chromosome region, candidate 8 | Nucleus | Transcription regulator | 79800 | 1.042 |
| OFD1 | Oral-facial-digital syndrome 1 | Plasma membrane | Other | 8481 | 1.021 |
| ZNF141 | Zinc finger protein 141 | Nucleus | Transcription regulator | 7700 | 1.015 |
| SH3BP2 | SH3-domain binding protein 2 | Cytoplasm | Other | 6452 | 0.982 |
| PIK3IP1 | Phosphoinositide-3-kinase interacting protein 1 | Unknown | Other | 113791 | 0.961 |
| CDKN1A | Cyclin-dependent kinase inhibitor 1A (p21, Cip1) | Nucleus | Kinase | 1026 | 0.943 |
| MEF2A | Myocyte enhancer factor 2A | Nucleus | Transcription regulator | 4205 | 0.929 |
| TP53INP1 | Tumor protein p53 inducible nuclear protein 1 | Nucleus | Other | 94241 | 0.927 |
| REV3L | REV3-like, catalytic subunit of DNA polymerase zeta (yeast) | Nucleus | Enzyme | 5980 | 0.880 |
| IGSF1 | Immunoglobulin superfamily, member 1 | Plasma membrane | Other | 3547 | 0.875 |
| ICA1 | Islet cell autoantigen 1, 69kDa | Cytoplasm | Other | 3382 | 0.861 |
| NEK11 | NIMA (never in mitosis gene a)- related kinase 11 | Nucleus | Kinase | 79858 | 0.857 |
| GDF15 | Growth differentiation factor 15 | Extracellular space | Growth factor | 9518 | 0.851 |
| AFF1 | AF4/FMR2 family, member 1 | Nucleus | Transcription regulator | 4299 | 0.848 |
| DNASE1L2 | Deoxyribonuclease I-like 2 | Extracellular space | Enzyme | 1775 | 0.837 |
| TFB1M | Transcription factor B1, mitochondrial | Cytoplasm | Transcription regulator | 51106 | 0.825 |
| MDM2 | Mdm2 p53 binding protein homolog (mouse) | Nucleus | Transcription regulator | 4193 | 0.816 |
| NOSTRIN | Nitric oxide synthase trafficker | Cytoplasm | Transcription regulator | 115677 | 0.783 |
| ACER2 | Alkaline ceramidase 2 | Cytoplasm | Enzyme | 340485 | 0.778 |
| FRK | Fyn-related kinase | Nucleus | Kinase | 2444 | 0.764 |
| CSNK1G1 | Casein kinase 1, gamma 1 | Cytoplasm | Kinase | 53944 | 0.752 |
| PPM1D | Protein phosphatase, Mg2+/Mn2+ dependent, 1D | Cytoplasm | Phosphatase | 8493 | 0.750 |
| ZKSCAN3 | Zinc finger with KRAB and SCAN domains 3 | Nucleus | Transcription regulator | 80317 | 0.693 |
| GCAT | Glycine C-acetyltransferase | Cytoplasm | Enzyme | 23464 | 0.681 |
| BTG2 | BTG family, member 2 | Nucleus | Transcription regulator | 7832 | 0.666 |
| TNFSF9 | Tumor necrosis factor (ligand) superfamily, member 9 | Extracellular space | Cytokine | 8744 | 0.666 |
| PGF | Placental growth factor | Extracellular space | Growth factor | 5228 | 0.656 |
| SESN2 | Sestrin 2 | Cytoplasm | Other | 83667 | 0.656 |
| MLL5 | Myeloid/lymphoid or mixed-lineage leukemia 5 (trithorax homolog, Drosophila) | Nucleus | Enzyme | 55904 | 0.647 |
| LY75 | Lymphocyte antigen 75 | Plasma membrane | Other | 4065 | 0.639 |
| PDK1 | Pyruvate dehydrogenase kinase, isozyme 1 | Cytoplasm | Kinase | 5163 | 0.635 |
| EIF2AK2 | Eukaryotic translation initiation factor 2-alpha kinase 2 | Cytoplasm | Kinase | 5610 | 0.631 |
| XYLT1 | Xylosyltransferase I | Cytoplasm | Enzyme | 64131 | 0.628 |
| PLTP | Phospholipid transfer protein | Extracellular space | Other | 5360 | 0.604 |
| DOK1 | Docking protein 1, 62kDa (downstream of tyrosine kinase 1) | Plasma membrane | Other | 1796 | 0.584 |
| IFT74 | Intraflagellar transport 74 homolog (Chlamydomonas) | Unknown | Other | 80173 | 0.573 |
| TSPAN7 | Tetraspanin 7 | Plasma membrane | Other | 7102 | 0.554 |
| RHOBTB2 | Rho-related BTB domain containing 2 | Unknown | Enzyme | 23221 | 0.535 |
| SEMA3A | Sema domain, immunoglobulin domain (Ig), short basic domain, secreted, (semaphorin) 3A | Extracellular space | Other | 10371 | 0.521 |
| ZMAT3 | Zinc finger, matrin-type 3 | Nucleus | Other | 64393 | 0.521 |
| MTERF | Mitochondrial transcription termination factor | Cytoplasm | Transcription regulator | 7978 | 0.516 |
| TRIAP1 | TP53 regulated inhibitor of apoptosis 1 | Cytoplasm | Other | 51499 | 0.513 |
| RUFY3 | RUN and FYVE domain containing 3 | Plasma membrane | Other | 22902 | 0.511 |
