# Supplementary material for: Investigation of Radiation-induced Transcriptome Profile of Radioresistant Non-small Cell Lung Cancer A549 Cells Using RNA-seq
Source: PLoS One. 2013 Mar 22;8(3):e59319. doi: 10.1371/journal.pone.0059319 (PMC3606344; doi:10.1371/journal.pone.0059319)
Supplement: Table S2 — Identification of significantly down-regulated genes in irradiated radioresistant A549 cells using RNA-seq. (DOC) [file pone.0059319.s002.doc]

**Table S2**

Identification of significantly down-regulated genes in irradiated radioresistant A549 cells using RNA-seq

| **Gene** | **Gene name** | **Location** | **Type(s)** | **Entrez gene ID**  **for human** | **Ratio** |
| --- | --- | --- | --- | --- | --- |
| ICAM2 | Intercellular adhesion molecule 2 | Plasma membrane | Other | 3384 | -2.219 |
| TESK2 | Testis-specific kinase 2 | Nucleus | Kinase | 10420 | -1.908 |
| UCN | Urocortin | Extracellular space | Other | 7349 | -1.876 |
| LOXL3 | Lysyl oxidase-like 3 | Extracellular space | Enzyme | 84695 | -1.725 |
| CACNB2 | Calcium channel, voltage-dependent, beta 2 subunit | Plasma membrane | Ion channel | 783 | -1.623 |
| IFIT3 | Interferon-induced protein with tetratricopeptide repeats 3 | Cytoplasm | Other | 3437 | -1.521 |
| GPER | G protein-coupled estrogen receptor 1 | Plasma membrane | G-protein coupled receptor | 2852 | -1.507 |
| TMOD1 | Tropomodulin 1 | Cytoplasm | Enzyme | 7111 | -1.360 |
| ANK3 | Ankyrin 3, node of Ranvier (ankyrin G) | Plasma membrane | Other | 288 | -1.210 |
| STAT5A | Signal transducer and activator of transcription 5A | Nucleus | Transcription regulator | 6776 | -1.200 |
| BAI1 | Brain-specific angiogenesis inhibitor 1 | Plasma membrane | G-protein coupled receptor | 575 | -1.172 |
| TBXA2R | Thromboxane A2 receptor | Plasma membrane | G-protein coupled receptor | 6915 | -1.118 |
| SLC23A1 | Solute carrier family 23 (nucleobase transporters), member 1 | Plasma membrane | Transporter | 9963 | -1.070 |
| GRHL3 | Grainyhead-like 3 (Drosophila) | Nucleus | Other | 57822 | -1.048 |
| COL4A4 | Collagen, type IV, alpha 4 | Extracellular space | Other | 1286 | -1.025 |
| SUPT3H | Suppressor of Ty 3 homolog (S. cerevisiae) | Nucleus | Transcription regulator | 8464 | -0.955 |
| ZNF589 | Zinc finger protein 589 | Unknown | Other | 51385 | -0.943 |
| TRAPPC2 | Trafficking protein particle complex 2 | Cytoplasm | Other | 6399 | -0.866 |
| KLF11 | Kruppel-like factor 11 | Nucleus | Transcription regulator | 8462 | -0.830 |
| IL12A | Interleukin 12A (natural killer cell stimulatory factor 1, cytotoxic lymphocyte maturation factor 1, p35) | Extracellular space | Cytokine | 3592 | -0.805 |
| MORF4L1 | Mortality factor 4 like 1 | Nucleus | Other | 10933 | -0.764 |
| TFEB | Transcription factor EB | Nucleus | Transcription regulator | 7942 | -0.760 |
| SCMH1 | Sex comb on midleg homolog 1 (Drosophila) | Nucleus | Transcription regulator | 22955 | -0.756 |
| ST6GAL1 | ST6 beta-galactosamide alpha-2,6-sialyltranferase 1 | Cytoplasm | Enzyme | 6480 | -0.735 |
| CPE | Carboxypeptidase E | Plasma membrane | Peptidase | 1363 | -0.714 |
| HMGN5 | High mobility group nucleosome binding domain 5 | Nucleus | Transcription regulator | 79366 | -0.713 |
| ITGAM | Integrin, alpha M (complement component 3 receptor 3 subunit) | Plasma membrane | Other | 3684 | -0.713 |
| RPS21 | Ribosomal protein S21 | Cytoplasm | Other | 6227 | -0.707 |
| POLM | Polymerase (DNA directed), mu | Nucleus | Enzyme | 27434 | -0.696 |
| RPL37 | Ribosomal protein L37 | Cytoplasm | Other | 6167 | -0.693 |
| MT2A | Metallothionein 2A | Cytoplasm | Other | 4502 | -0.685 |
| ICK | Intestinal cell (MAK-like) kinase | Cytoplasm | Kinase | 22858 | -0.680 |
| SPC25 | SPC25, NDC80 kinetochore complex component, homolog (S. cerevisiae) | Cytoplasm | Other | 57405 | -0.667 |
| CCDC41 | Coiled-coil domain containing 41 | Unknown | Other | 51134 | -0.665 |
| CEACAM1 | Carcinoembryonic antigen-related cell adhesion molecule 1 (biliary glycoprotein) | Plasma membrane | Trans-membrane receptor | 634 | -0.664 |
| ZNF354A | Zinc finger protein 354A | Nucleus | Transcription regulator | 6940 | -0.649 |
| SMAD1 | SMAD family member 1 | Nucleus | Transcription regulator | 4086 | -0.634 |
| KBTBD4 | Kelch repeat and BTB (POZ) domain containing 4 | Unknown | Other | 55709 | -0.630 |
| OPHN1 | Oligophrenin 1 | Cytoplasm | Other | 4983 | -0.619 |
| ST7L | Suppression of tumorigenicity 7 like | Unknown | Other | 54879 | -0.604 |
| RPS10 | Ribosomal protein S10 | Cytoplasm | Other | 6204 | -0.600 |
| TRNT1 | tRNA nucleotidyl transferase, CCA-adding, 1 | Cytoplasm | Enzyme | 51095 | -0.600 |
| SCN1B | Sodium channel, voltage-gated, type I, beta | Plasma membrane | Ion channel | 6324 | -0.598 |
| AGAP2 | ArfGAP with GTPase domain, ankyrin repeat and PH domain 2 | Nucleus | Enzyme | 116986 | -0.590 |
| PLEKHO1 | Pleckstrin homology domain containing, family O member 1 | Plasma membrane | Other | 51177 | -0.590 |
| F3 | Coagulation factor III (thromboplastin, tissue factor) | Plasma membrane | Trans-membrane receptor | 2152 | -0.572 |
| CARD8 | Caspase recruitment domain family, member 8 | Nucleus | Other | 22900 | -0.569 |
| RPL12 | Ribosomal protein L12 | Cytoplasm | Other | 6136 | -0.567 |
| PTPRG | Protein tyrosine phosphatase, receptor type, G | Plasma membrane | Phosphatase | 5793 | -0.564 |
| MNT | MAX binding protein | Nucleus | Transcription regulator | 4335 | -0.561 |
| PDE11A | Phosphodiesterase 11A | Cytoplasm | Enzyme | 50940 | -0.561 |
| WHSC2 | Wolf-Hirschhorn syndrome candidate 2 | Nucleus | Other | 7469 | -0.558 |
| ROMO1 | Reactive oxygen species modulator 1 | Cytoplasm | Other | 140823 | -0.554 |
| ZNF367 | Zinc finger protein 367 | Nucleus | Transcription regulator | 195828 | -0.553 |
| RPS13 | Ribosomal protein S13 | Cytoplasm | Other | 6207 | -0.516 |
| SLC37A1 | Solute carrier family 37 (glycerol-3-phosphate transporter), member 1 | Plasma membrane | Transporter | 54020 | -0.516 |
